# A Longitudinal Brain Magnetic Resonance Imaging Study of Neuromyelitis Optica Spectrum Disorder

**DOI:** 10.1371/journal.pone.0108320

**Published:** 2014-09-26

**Authors:** Su-Hyun Kim, So-Young Huh, Jae-Won Hyun, In Hye Jeong, Sang Hyun Lee, AeRan Joung, Ho Jin Kim

**Affiliations:** 1 Department of Neurology, Research Institute and Hospital of National Cancer Center, Goyang, Korea; 2 Department of Neurology, Kosin University School of Medicine, Busan, Korea; 3 Department of Radiology, Research Institute and Hospital of National Cancer Center, Goyang, Korea; Medical University Vienna, Center for Brain Research, Austria

## Abstract

Brain involvement is commonly seen in patients with neuromyelitis optica spectrum disorder (NMOSD). However, little is known about the chronic changes of acute brain lesions on MRI over time. Here, our objective was to evaluate how acute brain MRI lesions in NMOSD changed on follow-up MRI. We reviewed the MRIs of 63 patients with NMOSD who had acute brain lesions and follow-up MRI over an interval of at least 3 months. Of the 211 acute brain lesions, 24% of lesions disappeared completely on T2-weighed images (WI) and a decrease in size ≥50% on T2-WI was observed in 58% of lesions on follow-up MRI. However, 47% of lesions revealed focal T1-hypointensity and, in particular, 18% showed focal cystic changes. Cystic changes were observed most commonly in corticospinal tract and corpus callosal lesions whereas the vast majority of lesions in the cerebellum, basal ganglia and temporal white matter resolved completely. MRI remission on T2-WI occurred in 82% of lesions, while approximately half of the lesions presented foci of T1-hypointensity, which may be considered a severe tissue injury over time. The extent of brain injury following an acute brain lesion in NMOSD may depend on the location of the lesion.

## Background

The discovery of the anti-aquaporin-4 (AQP4) antibody advanced our understanding of neuromyelitis optica (NMO). NMO is now considered a disease distinct from multiple sclerosis (MS) in which antibodies against the water channel AQP4 play a critical role. Based on determination of the highly specific anti-AQP4 antibody, the spectrum of neurological manifestations associated with NMO spectrum disorder (NMOSD) is broader than previously recognized. In recent years, brain involvement has been reported in the majority of patients with NMOSD [1]–[10]. Some NMOSD patients show asymptomatic brain lesions on brain magnetic resonance imaging (MRI) at onset and even present with brain symptoms as their first manifestation [8], [11]. Certain types of brain lesions are now regarded as characteristic of NMO. These lesions involve the periependymal areas with high AQP4 expression, the corticospinal tracts, and hemispheric white matter [1]–[10]. The characteristic brain abnormalities on MRI play an increasingly important role in differentiating NMOSD from MS. Despite this, most previous studies of NMOSD have described only acute lesions or have not demonstrated a distinction between the acute and chronic stages, and little is known about the chronic changes in brain MRI lesions over time. The affected regions of the brain are usually edematous, showing hyperintensity on T2-weighted images (WI) with or without hypointensity on T1-WI, and may show gadolinium enhancement on conventional MRI [4], [6]. However, T1- and T2-prolongation are considered non-specific, because they are seen in a wide range of pathologies, including edema, inflammation, gliosis, demyelination, and axonal loss [12]. Investigation of the changes in brain MRI lesions is important for gaining an understanding of the extent of injury and can provide additional evidence for distinguishing NMOSD from MS. Furthermore, it can provide important hints for disease monitoring in NMOSD. In this study, we evaluated how acute brain MRI lesions in NMOSD changed on follow-up MRI.

## Methods

### Ethical Statement

This study was approved by the NCC Institutional Review Board (NCC2014-0055). As this study was retrospective design and involved no more than minimal risk to the patients, IRB approved the waiver of obtaining informed consent. Patient records/information was anonymized and de-identified prior to analysis.

### Patients

Consecutive patients with NMOSD (definite NMO, using the 2006 revised diagnostic criteria [13], or the seropositive limited form of NMO [5], [14]) who attended the MS clinic of the National Cancer Center (NCC), Korea, from May 2005 to September 2013 were recruited. Anti-AQP4 antibody levels were measured using an enzyme-linked immunosorbent assay (ELISA) [15] and a cell-based assay (CBA) using a commercial slide kit (Euroimmun, Luebeck, Germany) [16]. Patients who had at least one acute brain lesion and follow-up brain MRI over an interval of at least 3 months were included. Of the 176 patients with NMOSD in our cohort, 52 were excluded due to normal brain MRI and 61 were excluded due to incomplete data (either initial or follow-up MRIs were not performed). We retrospectively analyzed the demographic and clinical findings, including age, gender, attack history, dates of MRIs, and disability, as measured using the Expanded Disability Status Scale (EDSS) score.

### Magnetic resonance imaging

All MRI scans were performed using a 1.5-T or a 3.0-T machine. Brain scans included T1-weighted imaging (WI), T2-WI, fluid-attenuated inversion recovery (FLAIR), and gadolinium-enhanced sequences. All evaluations were performed by two neurologists (S.H.K, and S.Y.H.) and one neuroradiologist (S.H.L) with consensus. For each lesion, we recorded the location, number, signal intensity, size, and presence or absence of gadolinium enhancement. Acute lesions were defined as newly developed or enhanced brain lesions on brain MRI within 2 weeks after an acute attack, including a brain attack, myelitis, and optic neuritis. When MRI scans were performed at acute attack, high dose steroids were given after the scan. Based on our previous study [6], lesions were classified according to their location as follows: hemispheric white matter lesion, lesions involving the corticospinal tracts (e.g., posterior limb of the internal capsule extending to the cerebral peduncle or the ventral pons), periependymal lesions surrounding the aqueduct and the third and fourth ventricles (e.g., diencephalon, most dorsal part of the brainstem and cerebellar peduncle), periependymal lesions surrounding the lateral ventricles (e.g., corpus callosum and white matter adjacent to the lateral ventricles), tegmentum of the upper brainstem and other areas (cerebellum, basal ganglia and cortical/subcortical regions). Hemispheric white matter lesions were classified according to their major locations: frontal lobe, parieto-occipital lobe, or temporal lobe. As multiple hemispheric white matter lesions were observed in the same patients, individual white matter lesions were counted. Lesions involving bilateral areas were counted as two lesions. The patterns of chronic change on MRI were judged by assessing the (1) change in size on T2/FLAIR images (complete or near complete resolution, decrease in size ≥50% or decrease in size <50% of initial lesions); the decrease in size of lesion was determined by the changes in the longest diameter and cross-sectional area of hyperintense lesion on T2/FLAIR scans. (2) change in signal intensity on T1-WI (isointense or hypointense relative to the surrounding tissue: in particular, lesions showing significant hypointensity on T1-WI and hyperintensity on T2-WI, similar to that of cerebrospinal fluid, were termed “cystic change”), (3) change in gadolinium-enhanced lesions (disappeared, newly appeared or persistently enhanced lesions), and (4) appearance of new lesions on T2/FLAIR images.

### Statistical analysis

The numbers of T1-hypointense lesions or cystic changes were compared between patients with and without recovery using Mann-Whitney test. Statistical analysis was performed using STATA SE version 10.0 software (StataCorp, College Station, TX, USA) and difference was considered statistically significant at p<.05.

## Results

In total, 63 paired acute and follow-up brain MRIs in 63 patients with NMOSD (58 women, 92%; 43 patients with definite NMO and 20 seropositive patients with limited NMO) were examined. Of them, 60 (95%) patients were seropositive for the anti-AQP4 antibody. The median age at disease onset was 27 (range, 6–54) years, and the median age at the time of acute brain lesion detection was 29 (range, 11–54) years. The median disease duration from disease onset to the first scan is 15 (range 0–127) months. The median interval between the dates of acute brain lesion detection and follow-up MRI was 21 (range, 3–121) months, and the median disease duration at the time of the follow-up MRI scan was 44 (range, 4–184) months. Sixteen patients had no additional attack between the acute and follow-up MRI. The 47 other patients had more than one relapse of optic neuritis or myelitis between the two scans. The time from the last relapse and the follow-up brain MRI were median 5 (range, 0–16) months. At the time of the follow-up MRI scans, 46 (73%) patients were on immunosuppressive therapy; rituximab (n = 20), mycophenolate mofetil (n = 14), azathioprine (n = 8) and mitoxantrone (n = 4).

Brain symptoms were associated with acute lesions in 55 (87%) patients, and asymptomatic acute brain lesions were observed when optic neuritis or myelitis occurred in eight patients. Brain symptoms included lethargy, confusion, dizziness, headache, intractable vomiting, hiccups, hemiparesis/hemiplegia, hemiparesthesia/hemihypesthesia, diplopia, ataxia, facial hemiparesthesia, facial palsy, dysarthria, dysphagia, and respiratory difficulties. All patients were treated intravenously with methylprednisolone (1 g for 5 days) for acute relapses, and seven patients with brain symptoms underwent subsequent plasmapheresis following steroid therapy due to insufficient responses. Of the 55 patients with brain symptoms, 51 (93%) showed marked or moderate recovery with improvement in function, while four (7%) patients showed limited or no improvement after acute treatment.

In total, 47 (75%) patients had multiple brain lesions at the time of acute attack, and 19 (30%) patients met the Barkhof criteria for dissemination in space. A median of three (range, 1–11) lesions were observed in patients. The total number of acute brain lesions was 211 (Tables 1, 2): (1) hemispheric lesions (72 [34%] in 30 [48%] patients), (2) lesions involving the corticospinal tracts (e.g., posterior limb of internal capsule extended to the cerebral peduncle or ventral pons: 47 [22%]) in 33 [52%] patients), (3) periependymal lesions surrounding the aqueduct and the third and fourth ventricles (e.g., diencephalon, dorsal-most part of the brainstem, cerebellar peduncle, and medulla: 53 [25%]) in 33 [52%] patients), (4) periependymal lesions surrounding lateral ventricles (e.g., corpus callosum and white matter adjacent to the lateral ventricles: 24 [11%]) in 24 [38%] patients), (5) tegmentum of the upper brainstem: 8 [4%]) in 8 [13%] patients), and (6) other areas: 7 [3%]) in 6 [10%] patients). In total, 72 hemispheric white matter lesions were observed in 30 patients: 38 were located mainly in the frontal lobe, 27 in the parietooccipital lobe, and seven in the temporal lobe. The mean longest diameter of T2-WI hyperintense white matter lesions was 29±12 mm, and 26 of 69 (35%) lesions showed a longest diameter of more than 30 mm.

**Table 1:** Lesion distribution and chronic changes in brain lesion size on T2-weighted images.

Number of lesions (*n* = 211)	Hemispheric white matter lesions (*n* = 72)			Lesions involving corticospinal tracts (*n* = 47)	Upper brainstem and surrounding aqueduct and the third and fourth ventricles (*n* = 53)	Corpus callosum and area around lateral ventricles (*n* = 24)	Tegmentum of upper brainstem (*n* = 8)	Other (basal ganglia, cerebellum and cortical/subcortical) (*n* = 7)
	F (*n* = 38)	P-O (*n* = 27)	T (*n* = 7)					
Decrease in size ≥ 50% on T2-WI or FLAIR images, n (%)	32 (84)	19(70)	2 (29)	34 (72)	17 (32)	13 (54)	6 (75)	0 (0)
Decrease in size <50% on T2-WI or FLAIR images, n (%)	4 (11)	8 (30)	0 (0)	9 (19)	5 (9)	10 (42)	2 (25)	0 (0)
Complete disappearance, n (%)	2 (5)	0 (0)	5 (71)	4 (9)	31 (59)	1 (4)	0 (0)	7 (100)

Abbreviations: F, frontal lobe; P-O, parieto-occipital lobe; T, temporal lobe; T2-WI, T2-weighed images; n, number.

**Table 2:** Lesion distribution and chronic changes in signal intensity of T1-weighted images.

		Hemispheric white matter lesions (*n* = 72)			Lesions involving corticospinal tracts (*n* = 47)	Upper brainstem and surrounding aqueduct and the third and fourth ventricles (*n* = 53)	Corpus callosum and area around lateral ventricle (*n* = 24)	Tegmentum of upper brainstem (*n* = 8)	Others (basal ganglia cerebellum and cortical/subcortial) (*n* = 7)
Acute lesions (*n* = 211)	Chronic stage	F (*n* = 38)	P-O (*n* = 27)	T (*n = *7)					
T1 iso (*n* = 54)	T1 iso, *n* (%)	4 (10)	6 (22)	6 (86)	8 (17)	21 (40)	3 (13)	2 (25)	4 (57)
	T1 low, *n* (%)	0 (0)	0 (0)	0 (0)	0 (0)	0 (0)	0 (0)	0 (0)	0 (0)
T1 low (*n* = 157)	T1 iso, *n* (%)	12 (32)	4 (15)	1 (14)	8 (17)	24 (45)	2 (8)	4 (50)	3 (43)
	T1 low, *n* (%) cystic change, *n* (%)	22 (58) 4 (11)	17 (63) 7 (26)	0 (0) 0 (0)	31 (66) 21 (45)	8 (15) 0 (0)	19 (79) 7 (29)	2 (25) 0 (0)	0 (0) 0 (0)

Abbreviations: F, frontal lobe; P-O, parieto-occipital lobe; T, temporal lobe; iso, iso-signal intensity; n, number.

The acute lesions showed high signal and heterogeneous intensities on T2-WI with blurred margins. Abnormalities in the supratentorial region were more conspicuous on FLAIR imaging. Low signal intensity on T1-WI was observed in 157 (74%) acute brain lesions in 53 (84%) patients. Acute T2-hyperintense lesions corresponded to areas of slightly increased or isointense signal intensity on diffusion-weighted images. In apparent diffusion coefficient (ADC) mapping, the T2-hyperintense lesions correlated with increased ADC values in 60 of 71 (85%) lesions. Evaluation of gadolinium enhancement was available in 173 lesions in 58 patients. Most acute brain lesions did not show enhancement on T1-WI; indeed, enhancement was observed in only 28/173 (16%) lesions, in which the most common patterns were faint enhancement (n = 21), nodular enhancement (n = 5), pencil-thin ependymal enhancement (n = 1), and meningeal enhancement (n = 1).

**Figure 1:**
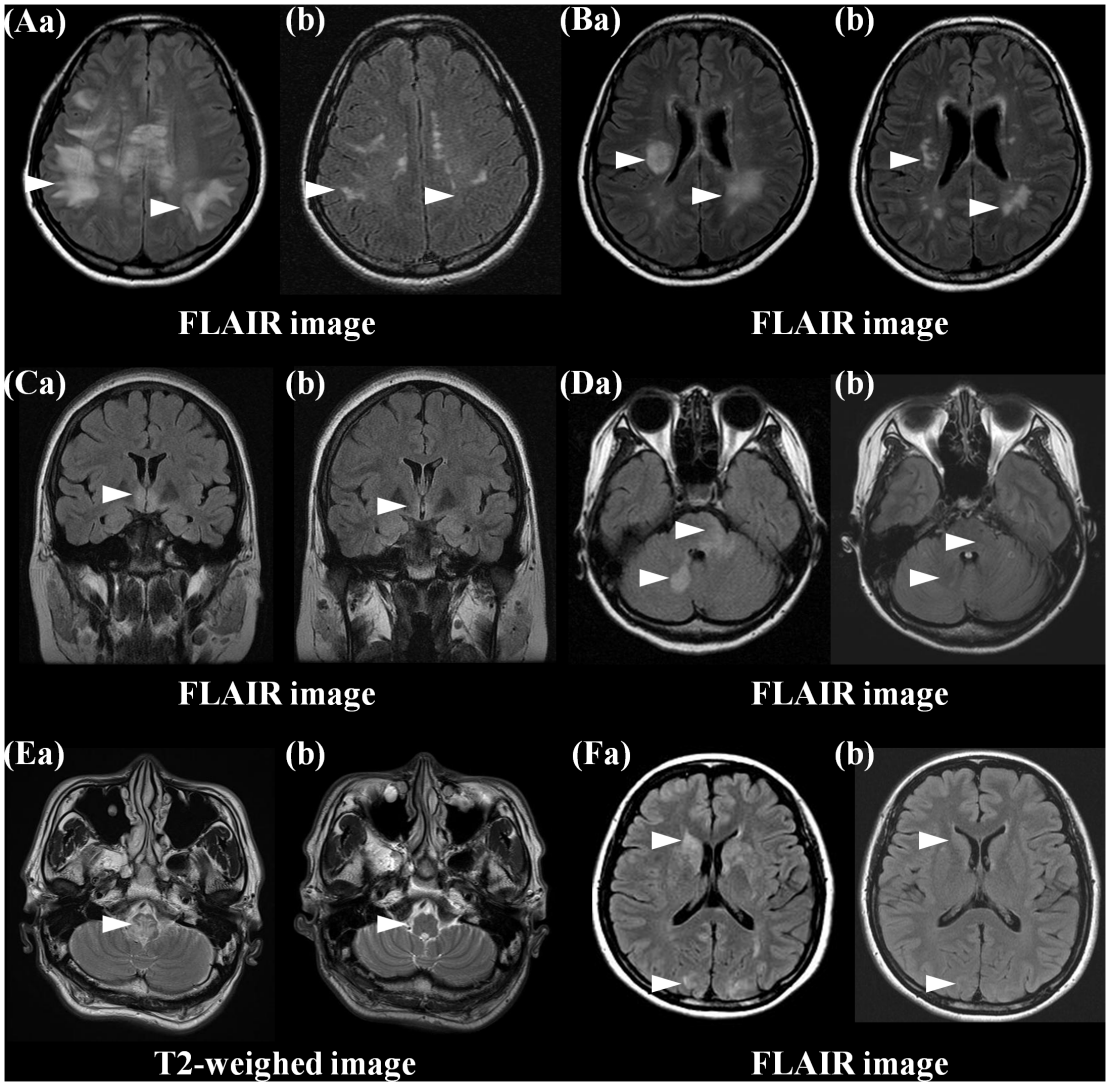
Most acute brain lesions in the white matter (Aa, Ba) showed a decrease in size and/or faded. Most remnant hyperintense lesions in hemispheric white matter on T2/FLAIR images were irregular in shape and located mainly in the subcortical/deep white matter (Ab, Bb). Acute hyperintense lesions in the bilateral hypothalamus (Ca) and around the fourth ventricle and cerebellum (Da, Ea), basal ganglia, and cortical/subcortical (Fa) on T2/FLAIR images resolved completely on follow-up brain MRI (Cb, Db, Eb, Fb).

On follow-up brain MRI at an interval of at least 3 months, most brain lesions showed a decrease in size and/or had faded. In total, 50/211(24%) lesions disappeared completely on T2/T1-WI in 34 (54%) patients. A decrease in size ≥50% on T2/FLAIR images was observed in 123/211 (58%) lesions in 39 (62%) patients, and a decrease in size <50% was observed in 38/211(18%) lesions in 18 (29%) patients. No increased lesion in size was observed on follow-up MRI. Remarkably, the majority of hemispheric white matter lesions revealed a decrease in size >50% on follow-up T2/FLAIR images. Most remaining lesions in the hemispheric white matter on T2/FLAIR images were irregular in shape, but no ovoid and located mainly in the subcortical and deep white matter (Fig. 1Ab, Bb). Of the 16 patients who had no clinical attack between the acute and follow-up MRI, none of them revealed a newly developed lesion.

**Figure 2:**
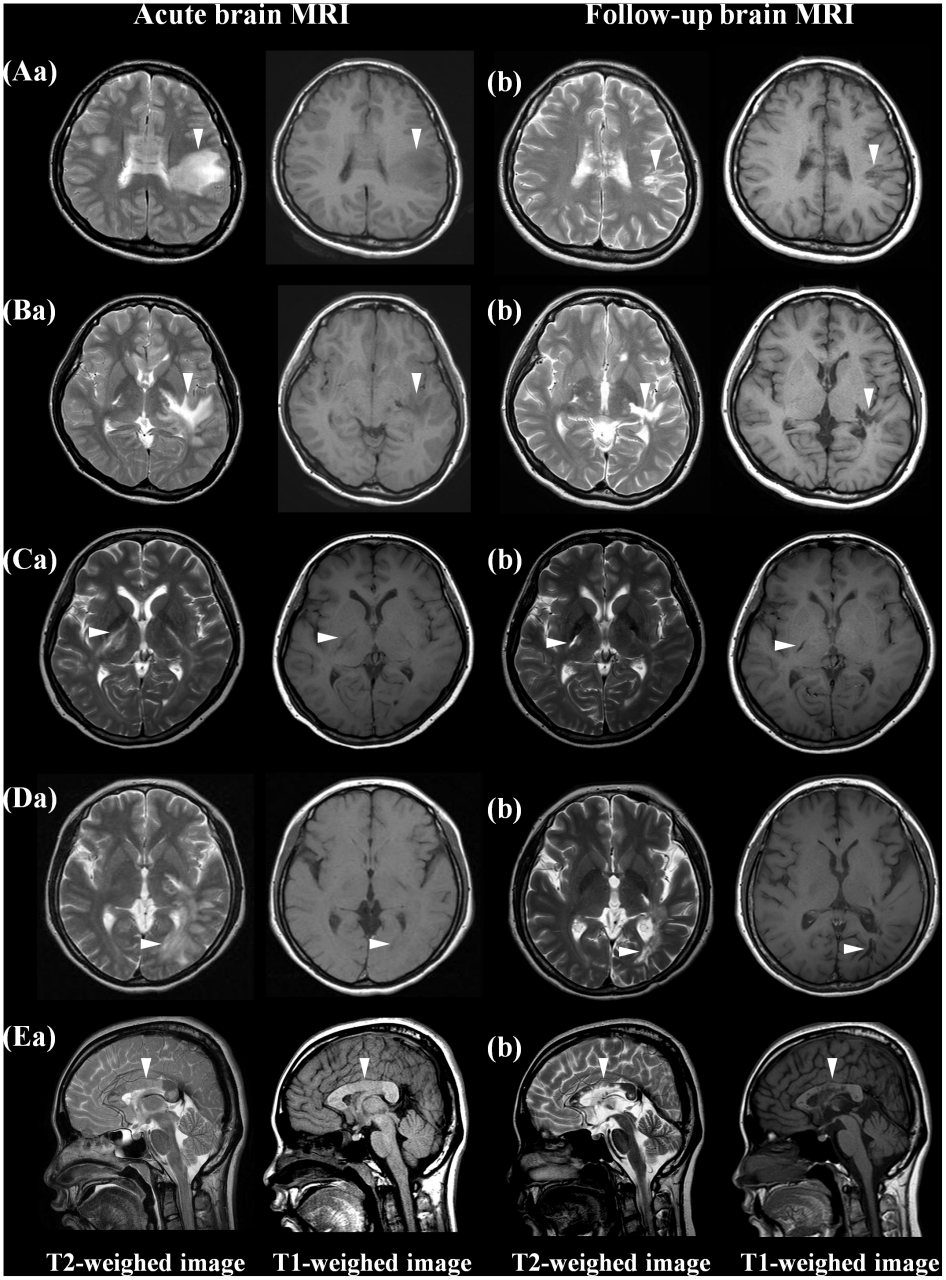
Chronic cystic changes of brain lesions on MRI. At the time of an acute brain attack, brain MRI showed multiple T2-hyperintense lesions with subtle T1 hypointensity in the frontal white matter (Aa), corticospinal tract (Ba, Ca), occipital white matter (Da) and corpus callosum (Ea). On follow-up MRI, all T2 hyperintense lesions were markedly decreased in size but revealed focal T1-hypointensity with cystic changes (Ab, Bb, Cb, Db, Eb).

While all 54 lesions with T1-isointensity at the acute stage remained T1-isointense on follow-up MRI, 99 of 157 (63%) lesions with T1-hypointensity at the acute stage showed focal T1-hypointensity on follow-up MRI in 30 patients. In particular, among them, 20 patients showed focal cystic changes in 39 lesions on follow-up MRI (Fig. 2). Among the 157 lesions with T1-hypointensity at the acute stage, 58 lesions (37%) became isointense on T1-WI later. Most lesions with T1-hypointensity on follow-up MRI also showed a marked decrease in size on T2-WI, while only 23/99 (23%) lesions with focal T1-hypointensity showed decrease in size a 50% on T2-WI. No persistent or reappearance of enhancement was observed on follow-up MRI. Patients with limited or no improvement from acute brain symptomatic attack revealed higher number of T1-hypointense lesions or cystic changes on follow-up MRI compared to patients with marked or moderate recovery (mean 3.4±1.5 vs. 1.5±1.7, p = .005).

**Figure 3:**
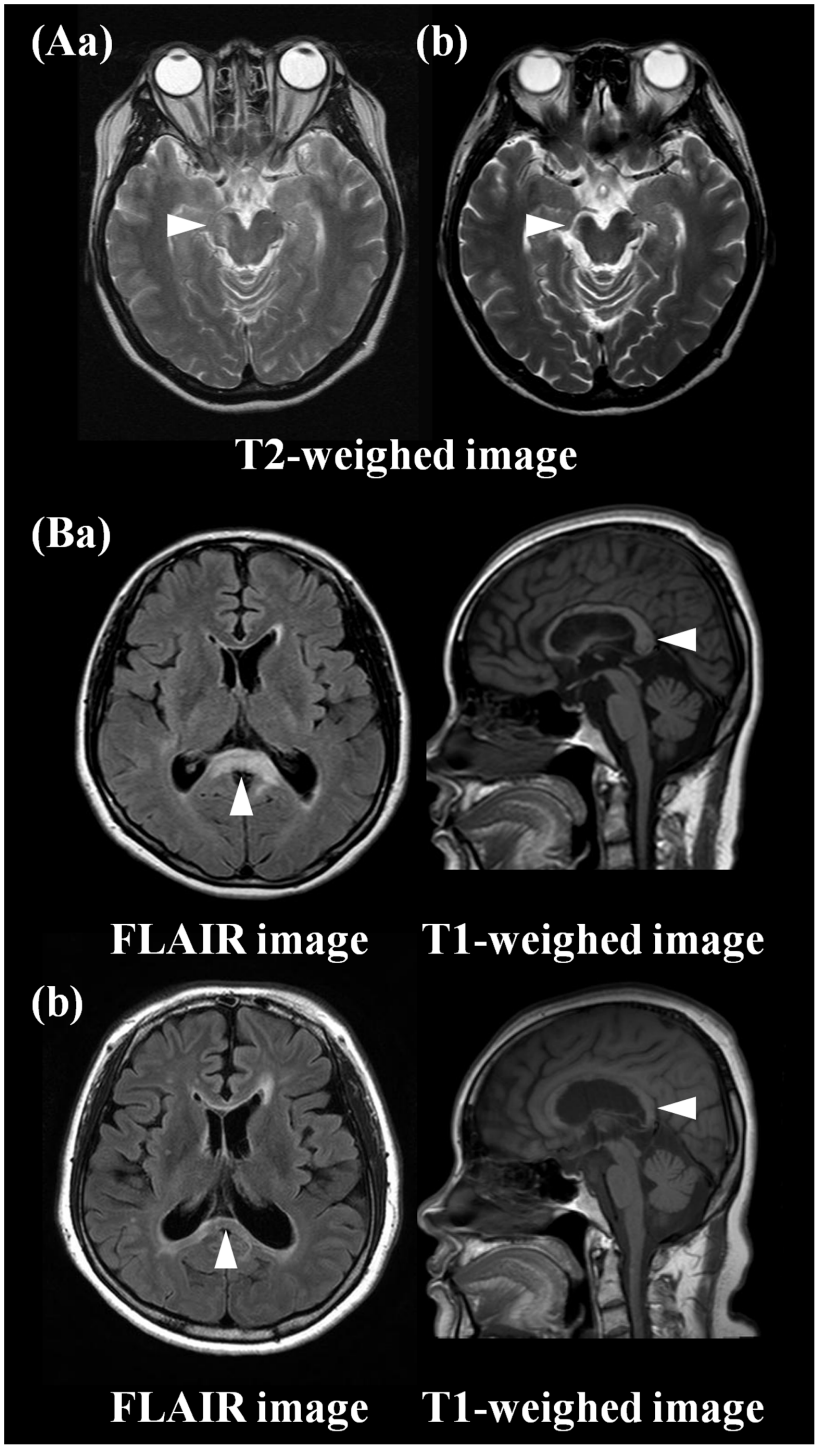
Focal atrophy on follow-up brain MRI. Acute lesions in crus cerebri (Aa) and corpus callosum (Ba) revealed focal atrophy with remnant T2-hyperintense lesions on chronic MRI (Ab, Bb).

Changes in MRI findings over time tended to show a characteristic pattern, depending on the location of the lesion (Tables 1, 2). Focal T1-hypointense lesions were observed most frequently in those involving the corpus callosum (79%), followed by the corticospinal tract (66%) and white matter lesions involving the parieto-occipital and frontal lobes (63% and 58%, respectively). In particular, cystic changes were seen most commonly in corticospinal tract lesions (45%), the corpus callosum (29%), and parieto-occipital white matter lesions (26%). Additionally, focal atrophy was observed in the corpus callosum (*n* = 3) and corticospinal tract (crus cerebri; *n* = 3) (Figure 3A, B). All lesions in the cerebellum, basal ganglia or diffuse cerebral/subcortical area, 71% of temporal white matter lesions, and 59% of lesions surrounding the aqueduct and the third and fourth ventricles resolved nearly completely (Figure 1 C, D, E, F).

## Discussion

In this study, we evaluated chronic changes in acute brain lesions on MRI in patients with NMOSD. More than half of the brain lesions decreased in size significantly on T2/FLAIR images, and a quarter of the lesions disappeared completely on follow-up MRI. No lesion increased in size, was newly developed, or reappeared with enhancement on follow-up MRI without a clinical attack.

While spinal cord or optic nerve attacks in NMOSD commonly result in severe clinical disability [17], clinical brain symptoms resolved markedly in 87% of our patients, consistent with the seemingly reversible brain lesions on MRI. The clinical and radiological resolution of brain attacks has been reported in previous case series [2], [18]–[20]. The mechanism of reversibility has been attributed to vasogenic edema, secondary to disruption of water transport during AQP4-directed autoimmune reactions [3], [7], [19]. Previously, we reported relatively well-preserved axonal structure and lack of necrosis in large cerebral lesions in a patient with NMOSD who showed significant resolution of extensive hemispheric lesions with clinical recovery on follow-up brain MRI [21].

In contrast, 99 (47%) and 39 (18%) of 211 chronic lesions presented focal T1-hypointensity and cystic changes, respectively, which could be considered consequences of severe tissue loss. This finding is also consistent with a previous case series in patients with NMO that indicated subsequent cavitational changes following acute attacks with extensive hemispheric lesions [10], [22]. Additionally, a recent study showed that callosal lesions were accompanied by cystic lesions more frequently in NMO than in MS [23]. Previous histopathological studies revealed extensive necrotic cavitary lesions associated with the loss of AQP4 immunoreactivity in the spinal cord lesions of NMO [24], as well as the formation of cystic lesions in the brain by a similar pathogenic mechanism [22].

The heterogeneous consequences, ranging from complete resolution to cystic changes in acute brain lesions on MRI, seemed to be related to the location of the lesion. Cystic changes were frequently observed in the lesions of corticospinal tract, corpus callosum, and white matter in the parieto-occipital lobe, whereas complete resolution, on MRI, was observed commonly in the cerebellum, basal ganglia, cortical/subcortical area, white matter in the temporal lobe, and periependymal area surrounding the third and fourth ventricles. Based on these results, it seems plausible that severe tissue injury following brain lesions may occur mainly in specific pathways connected with motor or visual systems. This hypothesis is consistent with a recent study in NMO that indicated selective occult structural damage in specific regions of the brain connected with the motor and visual systems [25].

An important question remains as to why the extent of injury differs from lesion to lesion. Previous NMO studies suggested that the variable vulnerability to AQP4 autoimmunity depended on the tissues involved. Binding of the anti-AQP4 antibody to AQP4 in astrocytes induces activation of complement, down-regulation of AQP4 and excitatory amino acid transporter 2 (EAAT2), and disruption of glutamate homeostasis [26]. Accordingly, the expression of complement-regulatory protein or EAAT2 in the involved tissue may affect selective vulnerability to AQP4 autoimmunity [9], [26], [27]. Furthermore, a positive association between NMO severity and AQP4-dependent complement activation in patient sera was reported [28], and the extent of complement activation was determined by the ratio of M1 to M23 proteins in the astrocytic membrane [29]. When the anti-AQP4 antibody binds to the extracellular epitope of AQP4, M1 is internalized completely, but M23 resists internalization and is aggregated into larger-order orthogonal arrays of particles that activate complement more effectively than M1 [29]. Thus, differences in the extent of injury in NMO brain lesions may be attributable to variable expression of complement-regulatory protein or EAAT2 and the extent of complement activation, which is determined by the M1:M23 ratio, depending on the tissue involved [26], [29].

The characteristics of chronic changes in brain lesions in NMOSD showed several features distinct from those of MS. The formation of new MS plaques is almost always associated with a focal area of contrast enhancement on T1-WI in patients with relapsing MS [30], and enhancement can also reappear in chronic lesions with or without a concomitant increase in size [31]. However, only 16% of acute lesions showed enhancement, and no newly appearing or persistently enhanced lesion was observed on chronic MRI in patients with NMOSD. The irregularly shaped remnant T2-hyperintense lesions in hemispheric white matter at the chronic stage were located mainly in the subcortical/deep white matter. Although there is no clearly defined criteria for “Dawson’s fingers” on MRI including details on borders, dimensions, and a distance from lateral ventricles [32], [33], Dawson’s finger-type lesion (wedge-shaped areas with a broad base to the ventricle and extensions into adjoining tissue in the form of finger-like processes or ampullae), which are seen typically in MS, were not seen in patients with NMOSD. The findings of the present study are consistent with those of Matthews et al., who suggested that patients with NMOSD do not exhibit Dawson’s fingers on brain MRI [34]. Recently, an ultrahigh field MRI studies suggested that MS white matter lesions were most frequently located in the periventricular white matter and centered by a small vein, but NMOSD lesions were predominantly located in the subcortical/deep white matter without central venule [35], [36]. Furthermore, in MS, fewer than 40% of newly formed lesions evolve into persistent or chronic black holes that show hypointensity on T1-WI compared with the surrounding tissue [37], [38], but cystic lesions rarely presented in the brain. Otherwise, 47% of chronic lesions in NMOSD presented focal T1-hypointense lesions and, in particular, 18% revealed cystic changes in specific areas, involving mainly the corticospinal tract, corpus callosum, and parieto-occipital white matter. These findings suggest that substantial irreversible brain tissue destruction may occur frequently in selected areas in NMOSD.

The present study is limited by its retrospective design, based on a single center in Korea, and the lack of serial assessment of MRI at regular intervals. Another limitation of our study is that the initial and follow-up MRI scans were not always performed on the same scanner. Additionally, a large proportion of patients with symptomatic brain lesions in our cohort may affect the frequent T1-hypointense lesions including cystic changes on follow-up MRI. Nevertheless, this study analyzed the pattern of chronic changes systematically in brain lesions in NMOSD in a relatively large cohort compared with previous case series. The findings of this study suggest that most acute brain lesions in NMOSD tend to result in good clinical and radiological recovery, but selective tissue loss may occur, mainly in specific pathways in the brain connected to the motor and visual systems. Additionally, patients with NMOSD showed a lack of continuous subclinical disease activity on brain MRI without relapse. These results provide additional information that will be valuable in understanding the pathology of brain lesions in NMOSD and in determining the differential diagnosis from MS. Further studies using serial MRI with short, regular intervals and multiple MR techniques are needed to better understand the pathophysiological mechanism(s) of brain lesions in NMOSD.
